# Beyond Averages: Chitosan Dispersity Affects the Bioactivity Reproducibility on In Vitro Maize (var. *Jubilee*) Germination

**DOI:** 10.3390/polym18091032

**Published:** 2026-04-24

**Authors:** Juan D. Giraldo, Ariana F. Peña, Claudia B. Briceño, Daniela Andrade-Acuña, Luis Aranibar, Karla A. Garrido-Miranda, Mauricio Schoebitz

**Affiliations:** 1 Laboratorio de Investigación en Biopolímeros (LIB), Escuela de Ingeniería Ambiental, Instituto de Acuicultura y Medioambiente, Universidad Austral de Chile, Sede Puerto Montt, Balneario Pelluco, Los Pinos s/n, Puerto Montt 5480000, Chile; luis.aranibar@uach.cl; 2 Departamento de Suelos y Recursos Naturales, Facultad de Agronomía, Campus Concepción, Universidad de Concepción, Concepción 4030000, Chile; arpena2018@udec.cl (A.F.P.); mshoebitz@udec.cl (M.S.); 3 Centro Superior de Ciencias Básicas, Universidad Austral de Chile, Sede Puerto Montt, Balneario Pelluco, Los Pinos s/n, Puerto Montt 5480000, Chile; claudia.briceno@uach.cl (C.B.B.); daniela.andrade@uach.cl (D.A.-A.); 4 Center of Waste Management and Bioenergy, Scientific and Technological Bioresource Nucleus, BIOREN-UFRO, Universidad de la Frontera, Temuco 4811230, Chile; karla.garrido@ufrontera.cl

**Keywords:** germination, chitosan, biostimulants, molecular weight, degree of acetylation, dispersity of the molecular weight, dispersity of the degree of acetylation

## Abstract

Chitosan bioactivity has been widely evaluated in seed germination; however, its effects remain inconsistent. Some studies report biostimulant effects, while others manifest inhibitory effects, and some show no effect on germination. A major factor contributing to this poor reproducibility appears to be the variation in the degree of polymerization (*X*), the molar fraction of acetylation (*f_A_*), and the chitosan concentration. However, it remains unclear whether controlling these parameters alone is sufficient to ensure consistent bioactivity in conventional polymeric chitosan samples. To elucidate this, maize seeds (*Zea mays L.* var. *Jubilee*) were soaked in chitosan solutions (pH 5) at concentrations ranging from 0.25 to 1.00% *w*/*w* for 4 h, as per the literature, to evaluate their effect on germination compared to a control (water). Nine chitosan samples were tested, differing in *X* (5558 to 17,161) and *f_A_* (0.07 to 0.33). After germination, several response factors were measured, including fresh and dry biomass, root number, and root and epicotyl length. The results showed that chitosans with higher *X* values (9134 to 17,161) inhibit germination, decreasing it by up to a value of 3% ± 6 at 1.00% *w*/*w*. Chitosans with a lower *X* (5694 ± 131) exhibited a reduced inhibitory effect (63% ± 6 to 100%) across all evaluated concentrations. None of the treatments showed biostimulation; instead, chitosan consistently delayed the germination rate compared to the control, confirming an inhibitory effect regardless of the final percentage. Nevertheless, while *X* and concentration establish the general inhibitory trends, considerable residual variability suggests that these factors alone are insufficient to ensure consistent bioactivity. A Gauge Repeatability and Reproducibility (R&R) analysis provides preliminary evidence suggesting that molecular heterogeneity, specifically dispersity (Ð*_X_*), is a key factor driving the observed inconsistencies and significantly affecting the reproducibility of the results within the scope of this study.

## 1. Introduction

Chitosan is a biopolymer with applications in several areas, including biomedicine, agriculture, water treatment, the food industry, and cosmetics [1]. This broad applicability is primarily attributed to its biocompatibility, biodegradability, low toxicity, and specific interactions with diverse living tissues (bioactivity). This versatility is a consequence of the wide range of possible chain conjugations enabled by its copolymeric nature, based on only two monomeric units: N-acetylglucosamine (GlcNAc) and glucosamine (GlcN). Three parameters define the polymeric structure of chitosan: the degree of polymerization (X), the molar fraction of acetylation (*f_A_*), and the pattern of acetylation (*P_A_*). Different combinations of these parameters can yield distinct physicochemical and biological properties. Regarding biological properties, some macromolecular configurations have been shown to act as biostimulants for plant cells [2].

Among these biostimulatory effects, its impact on seed germination has been evaluated in several plant species, particularly wheat, rice, and maize, due to their critical role in global food security [3]. Reported effects vary across studies, ranging from positive (P) to null (Nu) or adverse (A) outcomes (Table S1). However, several trends emerge from the literature: (i) the results depend on the crop species; (ii) lower degrees of polymerization (molar mass) enhance the biostimulant effect; (iii) the effect of the molar fraction of acetylation (degree of acetylation) on germination remains unclear; (iv) the chitosan concentration affects germination; (v) there is a poor characterization of the chitinous materials evaluated; and (vi) the mechanism of chitosan action that alters the performance of seed germination is not yet elucidated.

In relation to point (iv), the effect of concentration on germination is very ambiguous [4]. Which ranges of concentration are considered high, medium, or low? In the specific case of maize seeds soaked in chitosan solutions, the literature reports that concentrations of 0.01% *w*/*v* [5,6,7], 0.10% *w*/*v* [8], and 0.25% *w*/*v* had a null effect on germination [4,9], whereas concentrations of 0.50% *w*/*v* [9], and 1.00% *w*/*v* biostimulated and inhibited it [9], respectively. Conversely, soy and wheat seeds soaked in diluted chitosan solutions ranging from 0.001 to 0.008% *w*/*v* exhibited improved germination [10,11]. However, a slowdown in germination was observed in *Ageratum houstonianum* seeds soaked in even more diluted chitosan solutions at concentrations ranging from 0.00025 to 0.001% *w*/*v* [12]. But even more contradictory is the optimal concentration of 2.00% *w*/*v*, within an evaluation range of 1.00 to 2.00% *w*/*v*, in a study of biostimulation of germination of paddy seeds [13].

Concerning point (v), it remains evident that insufficient attention is given to reporting at least the degree of polymerization (*X*) and the molar fraction of acetylation (*f_A_*) of the chitinous material under study [2], despite long-standing debate on its importance [14,15,16,17,18]. **Our meta-analysis of the current literature (summarized in Table S1) reveals that 72% of the studies used chitinous samples with either no or insufficient characterization:** 47% reported no parameters, 16% reported only one, and 9.4% provided only broad ranges. This highlights a significant gap in the evaluation of chitosan’s effects on seed germination [19].

Among the Gramineae family (mainly wheat, rice, and maize, the world’s major cereals), maize presents positive (P), null (Nu), or adverse (A) responses to treatments with commercial chitinous materials in 56% of studies (Table S1). Interestingly, 44% of these studies used industrial samples from global suppliers such as Sigma-Aldrich (Merck), which offers three main chitosans with low (product no. 448869), medium (product no. 448877), and high (product no. 419419) molecular weights. This distinction is important because it allows the establishment of an average degree of polymerization range within which different effects have been observed, focusing on chitosan polymers rather than chitosan oligomers.

Regarding point (vi), the effect of chitosan polymers on seeds after a priming treatment—where seeds are soaked in chitosan solutions under controlled parameters such as time, pH, concentration, and others—remains not fully understood. The goal of this process is to improve seed germination performance and seedling vigor, as chitosan has been demonstrated to enhance germination rate, root and shoot length, biomass production, and other plant growth indicators. These enhancements are attributed to the fact that priming with chitosan triggers metabolic activation, stimulating cellular processes such as the de novo synthesis of proteins and nucleic acids, the production of antioxidants and adenosine triphosphate (ATP), the accumulation of phospholipids and sterols, DNA repair, and possibly other physiological processes. Specifically, it has been quantitatively demonstrated that chitosan treatment of maize seeds increases α-amylase and protease activities, thereby reducing starch and protein content (stored as reserves in the seed), increasing soluble sugars (a source of energy), and inducing de novo protein synthesis, thereby accelerating germination [4,7,20].

However, there is no clarity on how chitosan macromolecules interact with seed cellular membranes or on the molecular mechanism that confers bioactivity. The results reported in the literature (see Table S1) indicate that not all applications of chitosan improve germination performance; in some studies, null effects or germination inhibition are observed at the same concentration at which others show positive results. This poor reproducibility appears to be due to variation in the *X* and *f_A_*, or it may depend on the species or the variety of the seed evaluated and the application methodology; however, although these parameters do not vary, there is an intra-noise that affects the reproducibility of the essays, which does not guarantee that the application of a specific chitosan at a particular condition always provokes the desired bioactivity. This situation is documented by different authors who point out that it stems from the underlying heterogeneity of the chitosan samples [2,14,16,18,21,22,23].

The present study evaluated nine chitosan samples with different molar fractions of acetylation, grouped into three ranges of the degree of polymerization, to examine how these parameters, along with polymer dispersity and concentration, influence maize seed germination. The aim was to clarify the ambiguous results reported in the literature and provide guidance for the future development of chitosan-based bioproducts designed to enhance seed germination or other applications that rely on the predicted bioactivity of this biopolymer. This work does not intend to reveal the mechanism of action of the chitosan that confers its bioactivity, but provides quantitative evidence of the effects of variations in characterization parameters (i.e., *X*, *f_A_*, and Ð*_X_*) on the biological activity of chitinous materials.

## 2. Materials and Methods

### 2.1. Reagents and Equipment

Three industrial and nine laboratory-prepared (own-source) chitosan samples were used. The industrial samples corresponded to low molecular weight (LMW, product no. 448869/Sigma-Aldrich, Santiago, Chile), medium molecular weight (MMW, product no. 448877/Sigma-Aldrich), and high molecular weight (HMW, product no. 419419/Sigma-Aldrich). Laboratory-prepared chitosans are heterogeneous deacetylated samples using 40–50% (*w*/*w*) NaOH at temperatures between 90 and 120 °C, followed by a mechanochemical depolymerization process (mechanical milling for 10–60 min) starting from α-chitin (Sigma-Aldrich). The conventional chemical aspects of these treatments are discussed in Giraldo et. al. (2024) [24]. After processing, all samples were purified by washing with deionized water until a neutral pH was reached and then dried. As discussed in Section 4.1, these processes are inherently random; therefore, each batch was independently characterized via ^1^H NMR and GPC/SEC (Table 1) to establish its specific molecular identity, which we consider the only reliable descriptor for reproducibility in this study. Maize seeds, *cv. Jubilee F1* (minimum germination 80%), were obtained from Farmer’s (Chile). KOH and CH_3_COOH (Merck, Santiago, Chile) were used to adjust pH. Sterile distilled water was used in all experiments.

The following equipment was used: EQ573 analytic balance (Bel, Santiago, Chile), EQ207 magnetic stirrer (Velp, Santiago, Chile), T-710H pH-meter (Peack Instruments Inc., Santiago, Chile), VEMTICELL 55 ECO oven (MMM Group, Santiago, Chile), Incubator Q315M27 (Quimis, Santiago, Chile), laminar flow hood AC2-451 (Esco, Santiago, Chile), autoclave Q293-232DZXATOP (Quimis, Santiago, Chile), 400 MHz NMR spectrometer (Bruker, Madrid, Spain), and GPC/RID-10A/CTO-20A (Shimadzu, Madrid, Spain).

### 2.2. Characterization of the Chitosan Samples

Industrial and laboratory-prepared chitosan samples were analyzed by ^1^H NMR and SEC-RI to obtain their molar fraction of acetylation and degree of polymerization (Table 1). The former was determined according to Lavertu et al., without modification [27]. A brief description of the methodology is: chitosan samples (approximately 10 mg) were dissolved in a solution of 1.96 mL D_2_O and 0.04 mL DCl and stirred at room temperature until complete dissolution. ^1^H NMR spectra were acquired on a 400 MHz spectrometer at 70 °C. The experimental parameters included a single pulse sequence with solvent presaturation, a 90° pulse width (11 µs), a 6 s relaxation delay, and a 2 s acquisition time, for a total of 64 transients. To corroborate the methodology, the sample 419419 was measured twice, and the standard deviation of its *f_A_* was ±0.0035.

The degree of polymerization was measured using a mobile phase of 0.2 mol/L CH_3_COOH and 0.1 mol/L CH_3_COONa at 30 °C, according to the solvent system proposed by Wang et al. [28]. Briefly, the standards were prepared using a polysaccharide calibration kit (Pullulan standards, PL2090-0100, Varian, Spain) covering a molecular weight range from 180 to 800,000 Da. The mobile phase was filtered through a 0.45 µm filter. Both the chitosan samples (0.7 mg/mL) and the standards (2 mg in 4 mL) were sonicated for 30 min and filtered through a 0.22 µm filter prior to analysis. The injection volume was 100 μL, the flow rate was 0.8 mL/min, the temperature was 30 °C, and the wavelength was 690 nm. The calibration curve exhibited a coefficient of determination of R^2^ > 0.99.

### 2.3. Chitosan Solutions Preparation

The chitosan samples were dissolved in 1.00% *w*/*w* CH_3_COOH. After dissolution, a 10 or 50% *w*/*w* KOH solution was used to adjust the pH to 5, using minimum volumes of the reagent (a few drops). Chitosan solutions with concentrations of 0.25, 0.50, 0.75, and 1.00% *w*/*w* were made for each sample. The pH and concentration range selected are in accordance with the literature, where bioactivity has been reported in maize seeds [4,9,29]. The initial pH of the chitosan solutions ranged between 3.4 and 4.1. The adjustment to pH 5.0 using concentrated KOH is a standard procedure in the literature to ensure both polymer solubility and seed physiological compatibility, without introducing significant variations in ionic strength or salinity stress.

### 2.4. Treatment of the Seeds with Chitosan

Maize seeds (*Zea mays L.*, var. *Jubilee*) were disinfected with a 0.80% (*v*/*v*) NaClO solution (150 seeds per 200 mL) for 10 min. Seeds were then washed six times with sterile distilled water (100 mL) and dried on sterile absorbent paper. Subsequently, 30 randomly selected seeds were soaked in a determined chitosan solution (50 mL at pH 5) or a control (sterile distilled water) for 4 h. After soaking, 10 seeds were transferred to sterile Petri dishes (Ø 10 cm) containing sterile qualitative filter paper of the same diameter, moistened with 2 mL of sterile distilled water. Petri dishes were incubated at 28 °C in the dark for 8 days. Each 24 h, 0.50 mL of sterile distilled water was added to the filter papers as needed to maintain humidity. All treatments were performed under a laminar flow hood. This procedure aligns with the literature regarding maize seed priming with chitosan [6,7]. The decision to use sterile distilled water as a control is based on the study by Chen-xia et al. [29], which found no significant differences in the germination energy of maize seeds at pH 5.1 and 6.4. Additionally, the majority of the literature consulted on chitosan seed priming uses water as a control (see literature from Table S1). The pH of the chitosan solutions was adjusted to 5.0 immediately before use. During the 4 h soaking period, the solutions remained stable with no visible changes.

### 2.5. Monitoring Response Factors

The experimental days were labeled as day 0, 1, 2, 3, 4, 5, 6, 7, and 8, with day 0 corresponding to measurements taken before incubation. The period between day 0 and day 4 represents the minimum time required to observe the first sprouts (1.5 to 2.0 mm) and initiate germination recording [7]. Direct measurements of the following response factors were conducted over this period: (i) fresh biomass (day 0, and days 4 to 8), and (ii) number of germinated seeds (days 4 to 8). Additional measurements were performed at day 8 on germinated seeds, including: (i) number of roots, (ii) root length, (iii) epicotyl length, and (iv) dry biomass after drying at 70 °C for 24 h. Germination percentage was calculated using Equation (1).

(1) $$Germination\left(\%\right)=\frac{number\;of\;germinated\;seeds}{total\;number\;of\;seeds}\times 100$$

**Figure 1:**
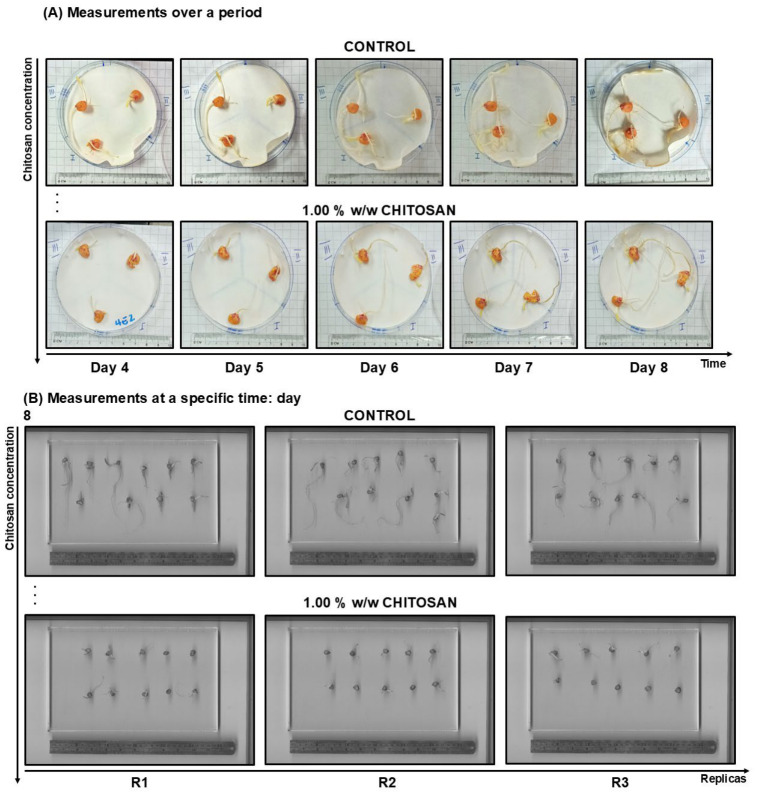
Examples of response factor measurements: (**A**) over a period, and (**B**) at a specific time. In (**A**), only three seeds are shown in a Petri dish to illustrate the progression of root and epicotyl growth. For quantification analysis, 10 seeds per Petri dish were used (see Section 2.3).

To clarify the methodology, Figure 1A illustrates the progression of seedling growth over time, while Figure 1B presents measurements taken at a specific time point, including the number of roots, root length, and epicotyl length. Manual measurements were performed for these parameters. All treatments and measurements were performed in triplicate.

### 2.6. Statistical Analysis

Response factors were evaluated using an analysis of variance (ANOVA) at a significance level of 0.05, followed by Tukey’s multiple comparison test to ascertain significant differences. The repeatability and reproducibility analysis of variance (Gage R&R analysis) was conducted using the add-in for Excel by Real Statistics using Excel (version Rel 9.6.1). Fresh and dry biomass, number of roots, root length, epicotyl length, and germination values were expressed as mean ± standard deviation. For germination, a qualitative measure scored as germinated (1) or non-germinated (0), and the standard deviation was calculated based on averages per Petri dish (*n* = 3). Regarding the inter-laboratory assay (Section 3.4), identical seed lots and reagent batches were used across both locations to ensure consistency and minimize uncontrolled variables.

## 3. Results

All samples were characterized using a consistent methodology, enabling direct comparison and establishment of a classification based on the degree of polymerization and molar fraction of acetylation (Table 1). The laboratory-prepared samples span the full range of degree of polymerization and acetylation fractions observed in the Sigma-Aldrich samples, providing a basis to elucidate the varying results reported in the literature (Table S1).

### 3.1. Effect on Germination

**Figure 2:**
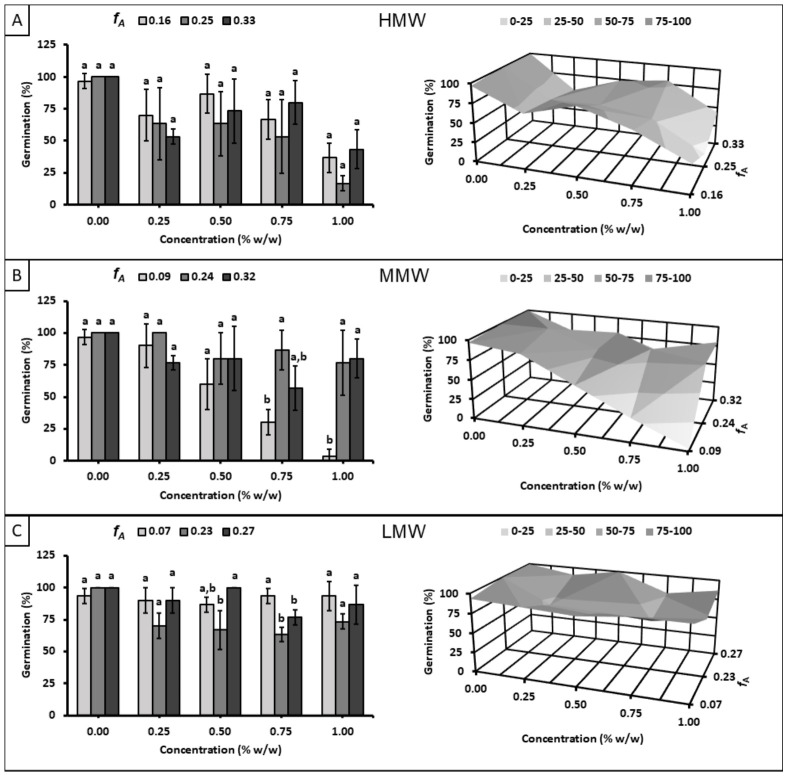
Effect of chitosan solutions with different molecular weight ranges on maize seed germination at day 8: (**A**) high molecular weight (*X*: 15353 ± 1922), (**B**) medium molecular weight (*X*: 9733 ± 556), and (**C**) low molecular weight (*X*: 5694 ± 131). Different letters indicate significant differences between *f_A_* according to Tukey’s test at *p* ≤ 0.05.

Figure 2 illustrates the effect of the different chitosan samples on germination at day 8. Two general trends were observed: (i) higher degrees of polymerization led to a greater inhibition of germination, and (ii) the observed inhibition increased with concentration within the medium-to-high molecular weight range. Within each molecular weight subset (HMW, MMW, or LMW), the effect on germination varied with the molar fraction of acetylation. HMW and LMW subsets exhibited the strongest inhibition at intermediate *f_A_* values, whereas the MMW subset showed minimal inhibition at lower *f_A_* values. Across all subsets, LMW samples caused the least inhibition across the evaluated *f_A_* and concentration ranges, with germination percentages ranging from 63 ± 6 to 100%. In contrast, the HMW subset, at the highest concentration and intermediate *f_A_*, reduced germination to 17% ± 6, while the MMW subset, at the highest concentration and lowest *f_A_*, reduced germination to a minimum of 3% ± 6.

The particularities observed in Figure 2 provide the primary explanation for the limited reproducibility of chitosan-based applications across different fields where its bioactivity is assessed [14,16,17,21,30]. In accordance with the aim of this study, it would be imprudent to assert that, within a MMW range, the trend of the *f_A_*-inhibitory effect on maize seed germination will invariably align with the observations in Figure 2B. Correlation with additional parameters is essential, as the reported *f_A_* and *X* values represent averages.

As shown in Table 1, the dispersity indexes (Ð*_X_*) of the degree of polymerization in the MMW subset are higher than in the other subsets. Notably, the *X* and Ð*_X_* values of the sample exhibiting the strongest inhibitory effect are the highest within the subset, consistent with the trend of increased inhibition with higher *X* values. This indicates that the Q09M sample contains a mass fraction of macromolecules with an *X* value exceeding 9733 ± 556. Conversely, the *f_A_*-dependent inhibitory trends observed in the HMW and LMW subsets did not show significant differences (*p* ≤ 0.05) across the evaluated *f_A_* values at most concentrations. A similar behavior was observed in the 0.00 to 0.50% *w*/*w* range within the MMW subset.

It is appropriate to note that since all chitosan samples were derived from the same chitin source and through a standardized process, the influence of impurities or variations in the pattern of acetylation (PA) is considered negligible across the set. Consequently, the observed biological responses—including particularities such as those in Q09M—are discussed based on the polymer’s intrinsic molecular parameters rather than external contaminants.

**Figure 3:**
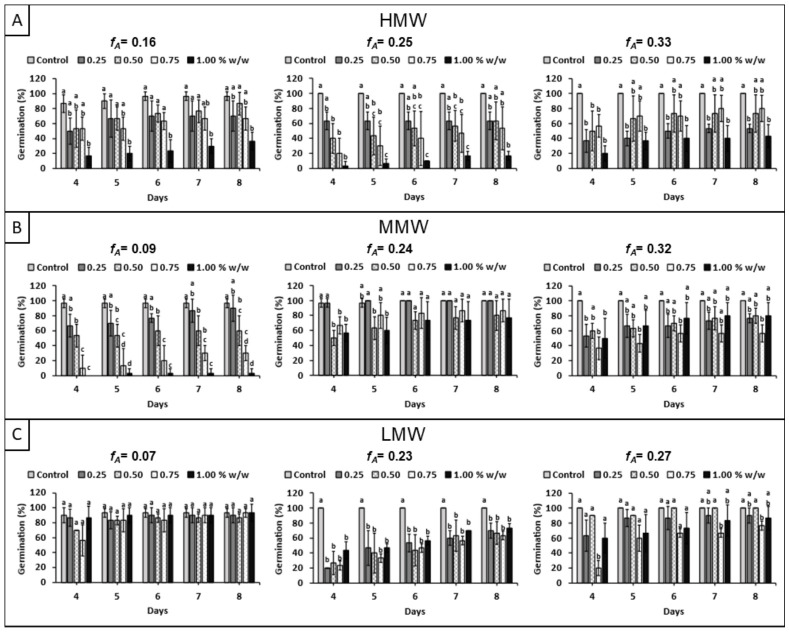
Effect of chitosan solutions with different molecular weight ranges on maize seed germination: (**A**) high molecular weight (*X*: 15,353 ± 1922), (**B**) medium molecular weight (*X*: 9733 ± 556), and (**C**) low molecular weight (*X*: 5694 ± 131). Different letters indicate significant differences among concentrations according to Tukey’s test at *p* ≤ 0.05.

Figure 3 shows the effect of different chitosan samples on maize seed germination rate. A general trend is that chitosan application delays germination, even in the seed sets that achieved a high degree of germination. However, increasing concentration does not consistently lead to higher inhibition in all samples. In the LMW subset, despite being the most homogeneous in both *X* and Ð*_X_*, the Q07L sample shows almost no inhibitory effect on germination rate, unlike Q23L and Q27L. This suggests that *f_A_* may indeed affect seed germination rate, a relationship that is not clearly observed in Figure 2 [31,32]. Furthermore, as *f_A_* approaches zero, its dispersion in the sample (Ð*_fA_*) is expected to decrease. Consequently, germination under the Q07L treatment was more homogeneous for all concentrations [14,16].

Figures S1 and S2 show that there were no significant differences in fresh or dry biomass throughout the growth period between control seeds and those impregnated with chitosan. The average biomass of untreated seeds (initial) was 0.29 g ± 0.01, while seeds with hydration for 4 h (day 0) with either water (control) or chitosan solution (regardless of concentration) reached 0.57 g ± 0.01. After 8 days of incubation, the average biomass was 0.60 g ± 0.04, and after drying at 70 °C for 24 h, it was 0.220 g ± 0.005. These averages and their respective standard deviations highlight the high homogeneity of the evaluated seeds and the treatment process. These also indicate that the increase in weight is principally due to retained humidity with an average of 49% *w*/*w* ± 2, and that chitosan solutions do not alter the hydration capacity of the seeds. This suggests that the effects observed on germination are not caused by the formation of a physical chitosan barrier that impedes water uptake, as proposed by other authors [7], but rather, it is more likely that the macromolecules of chitosan alter the ion-exchange capacity of the cellular seed membrane, as was observed in the study of Lyalina et al. (2023) [19].

### 3.2. Effect on Seedling Roots

**Figure 4:**
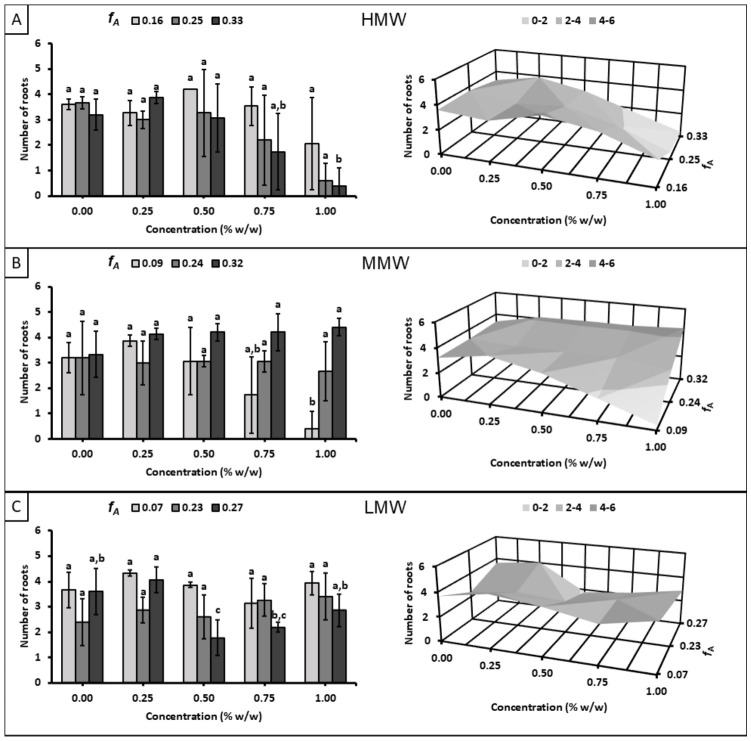
Effect of chitosan solutions with different molecular weight ranges on number of roots at day 8: (**A**) high molecular weight (*X*: 15,353 ± 1922), (**B**) medium molecular weight (*X*: 9733 ± 556), and (**C**) low molecular weight (*X*: 5694 ± 131). Different letters indicate significant differences among concentrations according to Tukey’s test at *p* ≤ 0.05.

Figure 4 shows the effect of chitosan solutions on the average number of roots per seedling at day 8, exhibiting trends similar to those observed in the germination results in Figure 2. In the HMW subset, higher concentrations decreased the average number of roots; in the MMW subset, this effect was observed specifically in the sample with the lowest *f_A_* (0.09); and in the LMW subset, root values remained relatively uniform across the concentration range evaluated. These results indicate that the number of roots is correlated with germination, i.e., a greater number of germinated seeds in the chitosan treatments yields an average root number closer to that of the control treatments, given that only germinated seeds were considered.

**Figure 5:**
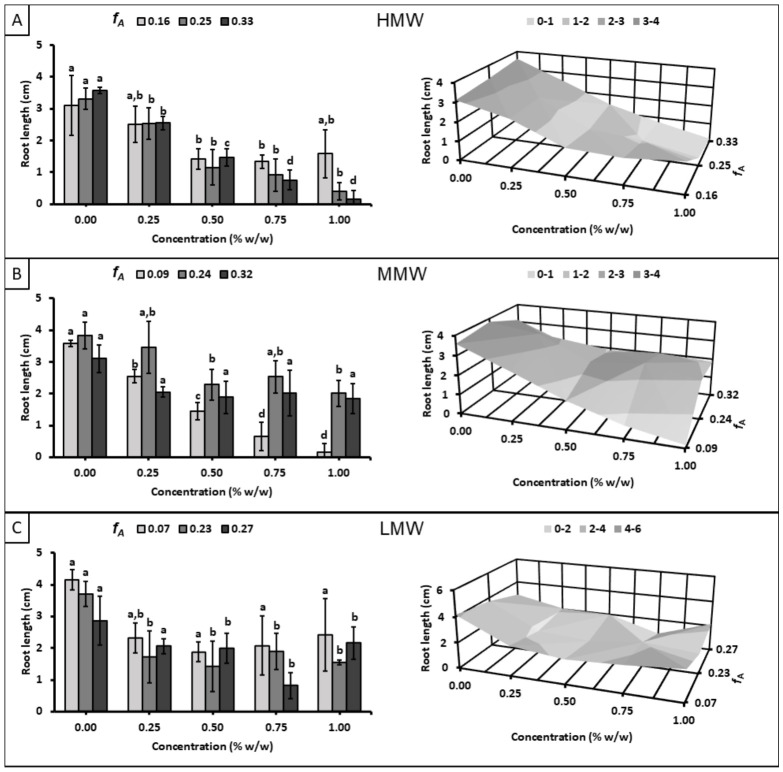
Effect of chitosan solutions with different molecular weight ranges on root length at day 8: (**A**) high molecular weight (*X*: 15,353 ± 1922), (**B**) medium molecular weight (*X*: 9733 ± 556), and (**C**) low molecular weight (*X*: 5694 ± 131). Different letters indicate significant differences among concentrations according to Tukey’s test at *p* ≤ 0.05.

Although no significant effect was observed on the average number of roots across the evaluated chitosan samples, Figure 5 shows an impact on the average root length. For all subsets (HMW, MMW, and LMW), average root length decreased with the chitosan application, and this trend became more pronounced as *X* and concentration increased. A noticeable delay in root growth was evident compared to the control, suggesting an inhibitory effect of the chitosan macromolecules. In this respect, no correlation between this behavior and *f_A_* was detected within the evaluated range.

### 3.3. Effect on Seedling Epicotyl

**Figure 6:**
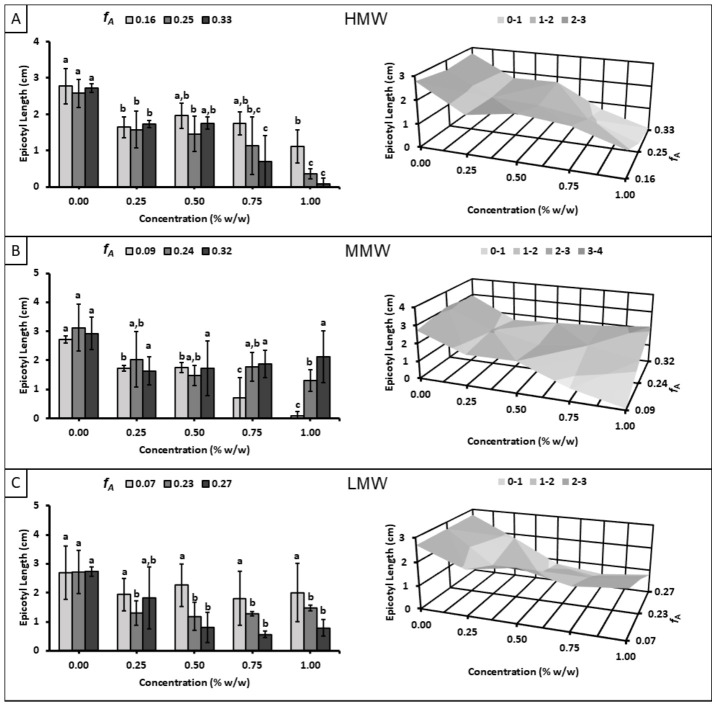
Effect of chitosan solutions with different molecular weight ranges on epicotyl length at day 8: (**A**) high molecular weight (*X*: 15,353 ± 1922), (**B**) medium molecular weight (*X*: 9733 ± 556), and (**C**) low molecular weight (*X*: 5694 ± 131). Different letters indicate significant differences among concentrations according to Tukey’s test at *p* ≤ 0.05.

Figure 6 shows the effect of chitosan solutions on epicotyl length at day 8. In general, it was observed that the application of chitosan decreased epicotyl length with respect to the control, and this reduction was directly proportional to chitosan concentration. In contrast, similar to the results observed for germination rate, the Q07L sample did not differ significantly from the control (*p* ≤ 0.05), suggesting that, at least at low degrees of polymerization, the inhibitory effect was less pronounced at lower *f_A_* values.

### 3.4. Reproducibility Essay

**Figure 7:**
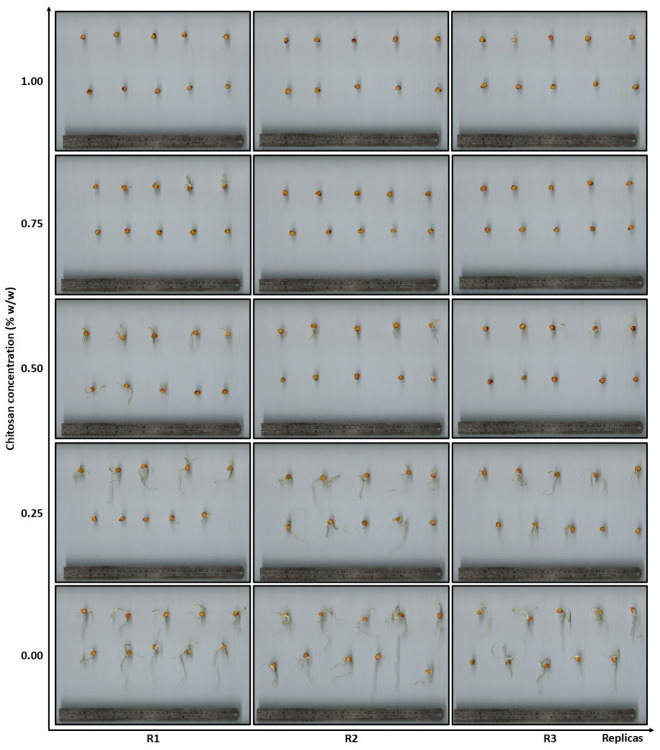
Scanning of seeds treated with the Q09M (*f_A_*: 0.09; *X*: 10,231; Ð*_X_*: 2.25) chitosan sample on day 8. Seeds with a sprout size between 1.5 and 2.0 mm were considered germinated. In some images, however, these small sprouts are not visible due to the selected zoom level.

The particularity observed in Figure 2 for sample Q09M (*f_A_*: 0.09; *X*: 10,231; Ð*_X_*: 2.25) shows a reduction in germination percentage to a minimum value of 3% ± 6. This value is lower than that obtained from the samples of the HMW subset and contradicts the general trend that germination inhibition decreases at lower molecular weight. This discrepancy suggests a potential error in the application of the methodology. Nevertheless, three independent replicas were conducted, and the results were consistent, as illustrated in Figure 7.

Moreover, although the averages indicate a general tendency across most evaluated monitoring factors, the substantial standard deviations observed in certain cases prevent the detection of a significant difference between means. For example, when comparing the effect of concentration in sample Q32M (*f_A_*: 0.32; *X*: 9134; Ð*_X_*: 2.22) across all monitoring factors, no significant differences were found. This contradicts the expected trend of an increased inhibition with increasing concentration.

These observations allow for the consideration of two explanations for the noted contradictions or particularities: (i) an inadequate application of the methodology by the laboratory technician, or (ii) an effect resulting from the dispersion (Ð*_X_* and Ð*_fA_*) of the samples. The latter explanation stems from the fact that all the chitosan samples evaluated are heterogeneous materials, consisting of partial fractions of macromolecules that vary in degree of polymerization and molar fraction of acetylation. Consequently, when a subsample is extracted from the primary sample, it may not accurately represent the original composition. This lack of representativeness resulting from the random selection of subsamples for bioactivity assessments at varying concentrations could potentially account for the observed inconsistencies.

To distinguish between both scenarios, an exploratory proof-of-concept essay with samples 419419 and Q07L were conducted in two different laboratories, at the Universidad de Concepción (UdeC) and Universidad Austral de Chile (UACh). The selection criterion was to include one sample with the highest dispersity (419419) and another with the lowest dispersity (Q07L). The underlying hypothesis was that lower dispersity in the degree of polymerization would result in more reproducible data, regardless of the laboratory or technician conducting the treatments.

**Figure 8:**
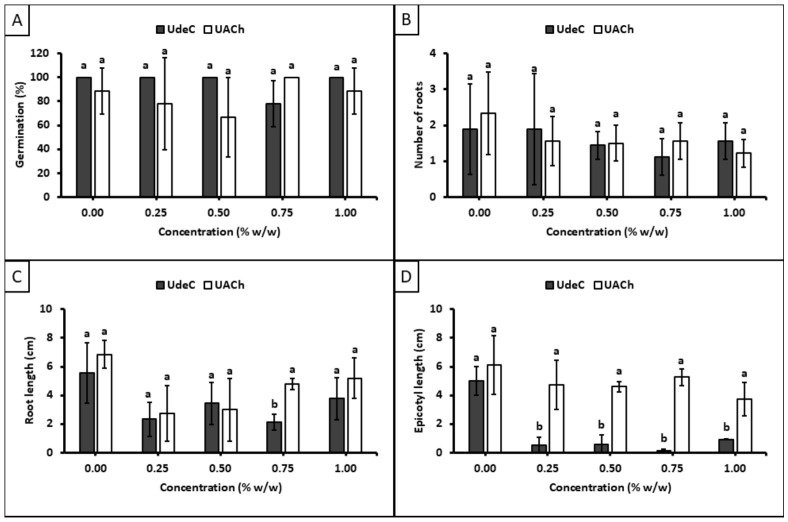
Effects of the treatments at day 8 with sample 419419 (Ð*_X_* = 2.53) on: (**A**) germination, (**B**) number of roots, (**C**) root length, and (**D**) epicotyl length. Different letters indicate significant differences between Universidad de Concepción (UdeC) and Universidad Austral de Chile (UACh) laboratories according to Tukey’s test at *p* ≤ 0.05.

Figure 8 illustrates the results of the treatments with sample 419419 (Ð*_X_* = 2.53). In terms of germination percentage (Figure 8A), it is noteworthy that, irrespective of concentration, nearly all seeds treated in the UdeC laboratory germinated at day 8, in contrast with the results obtained in the UACh laboratory. Although the Tukey-HDS test indicated no significant differences between the treatments performed in UdeC and UACh laboratories (as shown by the standard deviation bars), the differences in the magnitudes of standard deviations suggest a notable disparity in the repeatability of results at the level of individual seeds. Additionally, analysis of epicotyl length at day 8 (Figure 8D) revealed significant differences between the treatments performed at UdeC and UACh laboratories across all evaluated concentrations, indicating a considerable lack of reproducibility. The treatments conducted at UdeC showed marked inhibition of epicotyl growth, while those performed at UACh did not, when compared with the control.

**Figure 9:**
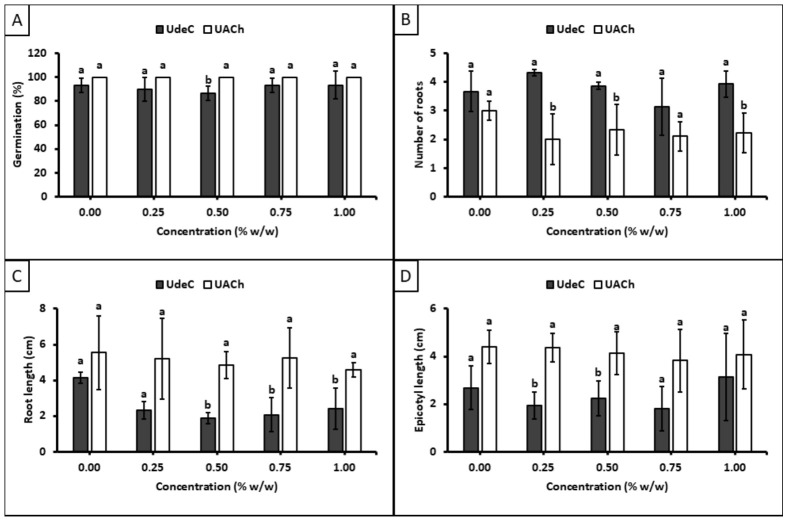
Effects observed in the intra-reproducibility essay of sample Q07L (Ð*_X_* = 1.87) on: (**A**) germination, (**B**) number of roots, (**C**) root length, and (**D**) epicotyl length at day 8. Different letters indicate significant differences between the Universidad de Concepción (UdeC) and Universidad Austral de Chile (UACh) laboratories according to Tukey’s test at *p* ≤ 0.05.

Upon evaluating sample Q07L (Ð*_X_* = 1.87), greater consistency between the two laboratories was observed (Figure 9). Regarding germination percentage (Figure 9A), similar to the results for sample 419419, one laboratory recorded a 100% germination for all treated seeds at day 8 (UACh), whereas the other laboratory (UdeC) did not reach this average. However, in this case, the extent of the standard deviations observed between the results from the two laboratories is minor, suggesting a minimal disparity in repeatability at the level of individual seeds. Furthermore, analysis of the other monitoring factors demonstrates a higher degree of reproducibility compared with the results obtained from sample 419419.

In order to quantify the variability introduced by either the operator (UdeC or UACh) or the samples (419419 or Q07L), a repeatability and reproducibility (R&R) analysis of variance (ANOVA) was conducted for each sample. Table S2, which summarizes the analysis of variance for sample 419419, demonstrates a part-to-part variation of 92.2% (where ‘part’ refers to treated seeds), exceeding the 30% threshold and suggesting that the selected components accurately reflect the true variation induced by the treatment. Conversely, the operator variation was 0.0%, below the 30% threshold, indicating minimal variability attributed to laboratory operations. These findings imply that variability is predominantly driven by the chitosan treatment rather than by the execution of the assay by the laboratory technician.

Table S3, which summarizes the analysis of variance for sample Q07L, shows a part-to-part variation of 98.9% and operator variation of 0.3%. These results indicate that variability is mainly driven by the treated seeds rather than the operator. Along with the analysis for sample 419419, the findings suggest that the laboratory technicians applied the methodology correctly and that the low reproducibility is potentially attributable to the nature of the chitosan samples. This indicates that sample dispersion (Ð*_X_*) may significantly affect measurement reproducibility within the cases evaluated.

The Total Gage R&R analysis (Tables S2 and S3), which quantifies variability within the measurement system, including both part-to-part and operator variation, indicates that the system is adequate. This conclusion is supported by the fact that Total Gage R&R values remain below the 10% threshold for both assays, specifically 7.8% for 419419 and 1.1% for Q07L, demonstrating that the employed methodology is acceptable. Notably, the higher value for 419419 indicates lower reproducibility compared to Q07L, consistent with its higher Ð*_X_* value. These findings provide **preliminary** support for the hypothesis that higher sample dispersity could reduce intra-sample reproducibility, thereby contributing to the variability observed across this specific set of experiments.

While a broader range of samples would be required to establish a formal correlation, these results highlight the risk of overlooking this factor in structure–activity studies. The obtained results indicate that classifying chitosan solely based on its degree of acetylation or polymerization may be insufficient for conducting a comprehensive structure–activity relationship study. At a minimum, it is highly recommended to determine the dispersity of the degree of polymerization. In the absence of a low Ð*_X_*, the biostimulation outcomes of industrial chitosans can be challenging to predict reliably, as substantial material heterogeneity is expected to result in low reproducibility.

## 4. Discussion

### 4.1. The Nature of the Dispersity of Conventional Chitosan

The degree of polymerization and molar fraction of acetylation of conventionally obtained polymeric chitosans can be influenced by several factors, including: (i) species, season of capture, and body part of the species from which the chitin is derived [33,34,35,36]; and (ii) the order of steps, duration, temperature, reagents, and concentrations used in the preparation method, including whether depolymerization or decoloration steps are applied [23,24,37]. Variations in chitin/chitosan sources affect crystallinity [36], type and quantity of impurities (e.g., protein, minerals, and heavy metals), moisture content [35], and macromolecule size [38], thereby inducing variability in the raw material [39]. This variability must be compensated for by adjusting standardized preparation parameters to achieve chitinous materials with the required average *X* and *f_A_* values [40]. However, even with such adjustments, there is no guarantee that chitosan from one batch will be identical to that from another. This is primarily due to the heterogeneous nature of the alkaline deacetylation of chitin, which occurs randomly and in parallel with alkaline hydrolysis (or oxidative-reductive depolymerization) in the presence of oxygen and high temperatures [41], which also occurs randomly [42].

The randomness observed in both reactions is primarily attributable to their occurrence within a solid–liquid system, in which chitin particles are suspended in a concentrated alkaline solution (~100 °C) that diffuses through the pores of the particles. During the initial phase of the reaction (0 to 30 min), diffusional limitations hinder the interaction of acetamide groups and glycosidic bonds with OH- ions, generating heterogeneity in the resulting materials (see the shrinking core model in the literature) [43,44]. At prolonged reaction times (>30 min), other factors are hypothesized to increase heterogeneity, including: (i) the formation of ‘free’ water that hydrates chitin macromolecules and (ii) the formation of a quasi-stable chitosan anion that impedes deacetylation [17,45,46]. This heterogeneity is reflected in the dispersion of both the degree of polymerization (Ð*_X_*) and the molar fraction of acetylation (Ð*_fA_*) [14]. Regarding *X*, even if depolymerization does not occur during the alkaline deacetylation [42,44,46,47], conventionally obtained chitosans always exhibit a Ð*_X_* > 1, as all chitins used as raw material are inherently polydisperse [38]. With respect to the pattern of acetylation (*P_A_*), given the random nature of the chemical treatments described above, it is expected to follow a predominantly random distribution [17,30,48,49,50,51,52,53,54,55,56]. It is necessary to explicitly state that, although the pattern of acetylation (*P_A_*) is a fundamental parameter influencing chitosan bioactivity, its direct experimental determination (e.g., ^13^C NMR, ^1^H NMR (500 MHz), HPLC, and mass spectrometry [48,49,50,51]) was not performed in this study. However, for conventionally prepared chitosans via alkaline deacetylation, the literature widely reports a predominantly random distribution of acetyl groups [30,52]. Therefore, in the following discussion, the *P_A_* is treated as a constant across all samples.

### 4.2. The Effect of Heterogeneity on the Bioactivity of Conventional Chitosan

Although some studies have evaluated the effects of variations in characterization parameters (i.e., *X*, *f_A_*, *P_A_*, Ð*_X_*, and Ð*_fA_*) on the biological activity of chitinous materials [30,57,58], only a few have considered Ð*_X_*, and none have addressed Ð*_fA_* [57,59,60]. These two parameters report the quality of the sample and indicate its level of heterogeneity of one sample (some authors consider that chitosan polymer samples with a Ð*_X_* < 2 have good quality [61]). At lower dispersity, the results from bioactivity assays of one specific chitosan polymer are more reliable. High dispersity limits the accuracy of analyses of the structure–activity relationship because it is not possible to determine which chitosan macromolecular species, or combinations of them, are responsible for the biological activity.

Several authors have expressed concern about the poor reproducibility of the bioactivity of chitosan polymers, attributing this to the high heterogeneity of the samples; however, no study has provided quantitative evidence for this [2,14,16,18,21,22,23]. In this respect, it is important to distinguish between inter- and intra-heterogeneity in the chitosan samples. The inter-heterogeneity refers to variations in the *X*, *f_A_*, *P_A_*, Ð*_X_*, and Ð*_fA_* between two or more samples, and the intra-heterogeneity relates to the dispersity of the degree of polymerization (Ð*_X_*) and molar fraction of acetylation (Ð*_fA_*) in one sample. In the first case, although *X*, *f_A_*, and *P_A_* are equal between two or more samples of conventional chitosan, if Ð*_X_* and Ð*_fA_* are higher than 1, there is no guarantee that the samples are homogeneous between them.

Numerous studies report the effects of inter-heterogeneity of conventional chitosan, indicating that no single chitosan exists and that differences in characterization parameters affect its bioactivity. One recent study reports partial consensus on the antimicrobial activity of chitosans, based on a careful analysis of 134 original papers. This points out that “the strongest antimicrobial activity was observed for chitosans with a low to medium average degree of polymerization. Larger polymers had lower activities, and chitosan oligomers were almost inactive. Less clearly, a trend was observed for decreasing activities with increasing average values of *f_A_*” [57]. Another study points out that the “inducing activity of systemic resistance in plants is more pronounced in chitosans with high average degrees of polymerization (>500) and intermediate average molar fractions of acetylation (0.2 to 0.5)” [2,62]. In the case of seed priming, the literature cited in Table S1 provides only partial consensus that chitosans with low average degrees of polymerization enhance germination. Specifically in maize, most studies report a null effect on germination at low concentrations, whereas only a few report biostimulant effects at medium concentrations or inhibition at high concentrations [4,5,6,7,8,63,64,65,66].

From the above, it is clear that **only partial consensuses on the biological activities of conventional chitosans can be extrapolated due to the poor homogeneity of the samples**, i.e., if Ð*_X_* and Ð*_fA_* are higher than 1, the comparison level between conventional chitosan samples to establish an effect as a function of average values of *X* and *f_A_* is subjected to the probability that macromolecules with equal values of *X* and *f_A_* in one sample are also present in the other samples. This probability increases as Ð*_X_* and Ð*_fA_* increase. The greater the probability that the same macromolecular species are present between the samples, the lower the level of comparability between them. This heterogeneity introduces noise, as evidenced by the standard deviations observed in this work, preventing detection of a significant difference (Tukey’s test) in the response factors due to variation in average values of *X* and *f_A_*. Additionally, as a methodological limitation, the use of a distilled water control prevents fully isolating the polymer’s effects from potential background pH or ionic contributions. According to the above, the results in this work showed the following partial consensus and particularities (Table 2).

However, a deeper view is presented in this work: the reproducibility of the bioactivity effects of conventional chitosans is not only affected by changes in the average values of *X* and *f_A_*, but also by intra-heterogeneity within the sample. Ergo, **applying the same sample of conventional chitosan under a specific condition does not always yield the same bioactive effect**. This is because high values of Ð*_X_* and Ð*_fA_* do not allow one to guarantee that a subsample has the same composition as the primary sample of conventional chitosan, especially if the subsample is subtracted without a preliminary quartering method. Additionally, directly subtracting subsamples from a primary sample will alter its composition, which, a posteriori, will alter the composition of future subsamples. The above affects the reproducibility of the essays, which does not guarantee that the application of a specific chitosan under a particular condition always provokes the desired bioactivity. This situation is documented by different authors who point out that it is an underlying effect of the heterogeneity of the chitosan samples [2,14,16,18,21,22,23].

This heterogeneity often leads to ambiguity in bioactivity studies involving chitinous polymer materials, as different studies often report contradictory results (see Table S1). These discrepancies are particularly concerning when specific bioactivities, such as biostimulation or biocidal action, are expected from the polymer. The resulting lack of reproducibility hinders the understanding of structure–function relationships in polymeric chitosans, complicates regulatory approval by government agencies, and limits the large-scale, routine application of products derived from these biopolymers [2,14,16,18,21,22,23].

### 4.3. How to Approach Conventional Chitosan Heterogeneity

Among all characterized samples, those provided by Sigma-Aldrich (Santiago, Chile) exhibited the highest *X* dispersity, which correlates with the poor reproducibility observed in the studies summarized in Table S1. The relevance of this parameter became evident in the present study, as subsets of samples with lower *X* dispersity (laboratory-prepared HMW and LMW samples) presented fewer discrepancies in monitoring factors and greater reproducibility in the intra-sample assay. According to Weinhold et al. [52], chitosans obtained from conventional methods do not fulfill the requirements for a structure–activity investigation without refinement, as they present a high Ð*_X_*. Refinement, in this context, refers to fractionation as a function of *X* in order to obtain chitosan products with lower Ð*_X_* values. However, this approach poses a technical challenge, as it is difficult to implement and finance at an industrial scale. This is evident given the absence of bulk commercial polymeric chitosans with Ð*_X_* values close to 1. Some pioneering studies have explored the feasibility of continuous processes for producing oligosaccharides through enzymatic depolymerization of chitosan, followed by fractionation via ultrafiltration [67,68,69]. Such strategies could be extrapolated to refine conventional chitosans. Nevertheless, as expected, the main problem with separation by ultrafiltration is the rapid membrane fouling due to the high viscosity of chitosan solutions, making this method inefficient for polymeric chitosan fractionation at an industrial scale.

Recent advances in alternative production processes for chitin, chitosan, and their oligomers provide some insights to overcome this limitation [33]. In particular, mechanochemical methods show remarkable potential for scalability and for producing chitinous materials with Ð*_X_* values closer to 1. Mechanochemistry also represents a promising alternative to produce oligomers or small polymers of chitin and chitosan with more consistent *X* distributions. Evidence suggests that higher milling energies (rotation frequencies) lead to more uniform particle sizes and molar masses (Ð*_X_* ~ 1.1) as milling time increases [70]. However, obtaining polymeric chitosans with molecular masses in the range of the samples used in this work, but with a lower Ð*_X_*, remains challenging, because mechanical forces applied to chitinous materials always provoke their depolymerization, which is contrary to achieving high degrees of polymerization.

Another strategy to address the poor reproducibility of the direct application of polymeric chitosan as solvated macromolecules with high Ð*_X_* and Ð*_fA_* values is its transformation into nanoparticles. Several studies have evaluated the biological activity of chitosan nanoparticles, demonstrating that they can improve seed germination, plant growth, and crop protection [71]. Research on maize seed priming comparing chitosan nanoparticles with chitosan solutions has shown that conversion to nanostructures increases bioactivity [7]. An additional advantage is that these nanoparticles could be formulated as nanocomposites that incorporate micronutrients (such as Cu, Zn, Fe, Si), thereby further enhancing biological activity [5,6,7,65,66]. This transformation into nanoparticles implies that non-ionized structures, rather than ionized macromolecules, interact with plant membranes, thus avoiding the polyelectrolyte range. This interaction allows the activation of natural hydrolases that produce oligomers, which are recognized by plant membrane receptors and elicit a positive biological response. In contrast, ionized macromolecules perturb membrane integrity, promoting harmful biological effects (commonly biocidal activity or phytotoxicity), which, in the context of this study, is observed as inhibition of germination.

However, most chitosan nanoparticles studied for agricultural applications are formed by physical rather than chemical forces. This is mainly favored because it requires milder conditions and preserves chitosan properties such as biocompatibility and biodegradability. While advantageous, these kinds of chitosan nanoparticles present a disadvantage that is not commonly documented: their structure is influenced by dissolution factors and ionic strength [72]. If these nanoparticles are applied through irrigation or spraying systems together with nutrient solutions, typically containing salts, their dissolution may occur. As a result, direct effects may not be exerted by the nanoparticles themselves but by solvated macromolecules or aggregates of the original chitosan. This scenario underscores the importance of considering the specific type of chitosan used for nanoparticle formation, and the present study may serve as a guide for such selection. For more insight into enhancing seed germination and plant growth through the application of chitosan nanoparticles, consult the recent review by Riseh et al. (2024) [71].

## 5. Perspectives and Future Directions

It is important to explicitly note that the mechanisms, theoretical models, and analytical frameworks proposed in the following section are hypotheses and extrapolations intended to guide future research. While they are grounded in the physicochemical principles discussed earlier, they are not direct experimental conclusions derived from the bioactivity or characterization data obtained in this study.

### 5.1. Aggregates, Interactions, and Mechanisms

In the present study, the biostimulation of seed germination through soaking in chitosan solutions involves the direct interaction of chitosan macromolecules with the vegetative cells of seeds, thereby exerting a direct effect [2]. This premise suggests that chitosan exhibits biological activity that is directly related to its molecular configuration. Consequently, the characterization parameters of chitosan are relevant, and their omission may lead to bias in experimental results. According to Wattjes et al. (2020), chitosan samples can be classified either as polymers with polyelectrolyte behavior (*f_A_* < 0.25) or as polymers with hydrophobic/hydrophilic behavior (0.25 < *f_A_* < 0.5) [30]. If the average degree of ionization (β) is determined at pH 5, as shown in Table 3, all the samples present a β > 0.5. Rinaudo et al. (1999) indicated that solubilization of chitosans begins at this threshold [73]. However, Giraldo et al. (2021) reported that for a high-molecular-weight sample with a *f_A_* of 0.31, complete solubilization occurs at β ≥ 0.66 [74]. Based on these findings, it is likely that the chitosan solutions applied to the seeds were, in some cases, only partially soluble systems with suspended aggregates. This may have led to greater heterogeneity among the macromolecules interacting with the seed cell walls, as not all the macromolecules within a sample would be fully solvated, even when sharing the same β value. In these partially soluble systems, the solvated macromolecules were probably more ionized than the β values reported in Table 3. This is attributed to the fact that the crystalline nature of the aggregates prevents the ionization of internal amine groups, thereby increasing the hydronium ion concentration in the medium and causing solvated macromolecules to have greater degrees of ionization than β.

For HMW chitosans, which are generally regarded as less soluble [75,76], this phenomenon probably intensifies electrostatic interactions between highly ionized (+) macromolecules and the negatively charged (−) pericarp of the seeds, thereby altering the ion-exchange capacity of the cellular seed membrane [19], and ultimately leading to greater germination inhibition via a phytotoxic effect. These assumptions are consistent with the results obtained: a decrease in the degree of polymerization reduces the inhibitory effect. It is reasonable to assume that, in the case of LMW chitosans, macromolecules are less ionized (closer to β) and therefore exhibit weaker electrostatic interaction with the seed pericarp. As shown in Figure 2, LMW chitosans either do not inhibit germination (*f_A_* 0.07) or only slightly inhibit it (*f_A_* 0.23 and 0.27). The observed differences in inhibitory effects across molar fractions of acetylation suggest greater homogeneity in *f_A_* for sample Q07L, which influences both its degree of ionization (closer to β) and its solubility, and consequently, its interaction with the seeds.

Even though, previously, experiments with a conventional chitosan with an *X* = 11,359 and *f_A_* = 0.31 at similar concentrations to those evaluated in this work (0.25% w ≈ 0.0010 mol/L; 0.50% w ≈ 0.0020 mol/L; 1.00% w ≈ 0.0040 mol/L) [74] showed that partial solubilized systems exist below β values of 0.66 through analysis of the transmittance at 600 nm, this measurement was not made for the chitosan samples evaluated in this work and the above described have to be assumed as a hypothesis that has to be corroborated through future analysis using analytical techniques such as UV-vis, DLS, reology, ζ-potential and others to determine the presence of aggregates and the interactions with the cellular seed membrane. These analyses, together with measurements of enzyme activity in soaked seeds [7], could allow us to establish the mechanism by which conventional chitosan solutions affect germination.

### 5.2. Conceptual Framework for Determining Ð_fA_

Although some studies have evaluated the effects of variations in characterization parameters (i.e., *X*, *f_A_*, *P_A_*, Ð*_X_*, and Ð*_fA_*) on the biological activity of chitinous materials [30,57,58], only a few have considered Ð*_X_* [59,60], and none have addressed Ð*_fA_*. This is because, while determining the dispersity of the degree of polymerization is now feasible, measuring the dispersity of the molar fraction of acetylation in chitosan polymers remains technically challenging [16]. To determine it, fractionation techniques must be applied; however, fractionation based solely on *f_A_* is difficult because other factors, such as acetylation pattern, degree of polymerization, and degree of ionization, are also involved [77]. Furthermore, proposed fractionation methods, including pH-induced precipitation [14], solvent-based precipitation [78], size exclusion chromatography (SEC) [60], and capillary electrophoresis [31,32], are affected by one or more of these parameters.

To understand the difficulty described above, a **conceptual approach for assessing** Ð*_fA_* could be helpful. A model case is a chitosan sample of nine macromolecules (M) with the following parameters of characterization: M1 (*X* = 12, *f_A_* = 0.500, *P_A_* = 1 (random)), M2 (*X* = 12, *f_A_* = 0.417, *P_A_* = 1), M3 (*X* = 12, *f_A_* = 0.333, *P_A_* = 1), M4 (*X* = 10, *f_A_* = 0.500, *P_A_* = 1), M5 (*X* = 10, *f_A_* = 0.400, *P_A_* = 1), M6 (*X* = 9, *f_A_* = 0.333, *P_A_* = 1), M7 (*X* = 6, *f_A_* = 0.500, *P_A_* = 1), M8 (*X* = 6, *f_A_* = 0.667, *P_A_* = 1), and M9 (*X* = 3, *f_A_* = 0.333, *P_A_* = 1). Without taking into account the X and PA, the grouping of the macromolecules that have the same *f_A_* results in five sets: A (M1, M4, M7), B (M3, M6, M9), C (M2), D (M5), and E (M8). As can be noted, the *f_A_* is independent of the *X*. Although Mnatsakanyan et al. (2013) defined the weight fraction of acetylation (*f_A-w_*), this one does not correlate with the number fraction of acetylation (*f_A-n_*) in the same distribution curve to determine the Ð*_fA_*, because two or more macromolecules with different molar masses could have the same *f_A_* [31,32]. It is more appropriate to use the equation proposed by Roberts et al. (1997) that relates the bulk sample *f_A_* with the highest value of molar fraction of acetylation (*f_A-max_*) of the isolated fractions to determine the Ð*_fA_* [14]. However, a change in this expression is necessary because it is probable that the distribution of the *f_A_* in heterogeneous samples will not be normal (as is in this example). For that reason, the bulk sample *f_A_*, which is commonly interpreted as the average *f_A_* of the sample, must be the median *f_A_* value of the sample, and the expression will be Ð*_fA_* = *f_A-max_*/*f_A-median_*. In this example, the Ð*_fA_* is 1.6.

Technically, the challenge is how to separate the macromolecules solely based on their *f_A_*. One option is to leverage the fact that the linear charge density of the chains correlates with their molar fraction of deacetylation (*f_D_* = 1 − *f_A_*), assuming complete ionization of all amino groups in one chitosan sample. Given that the linear charge density represents the quantity of net electric charge distributed per unit length of the chain, if the degree of ionization is equal to 1, macromolecules with the same *f_A_* will possess identical linear charge densities, irrespective of the value of *X*. In this scenario, the chains could be mobilized at different velocities as a function of the linear charge density under an applied electric field and then locally separated using an isoelectric focusing (IEF) technique.

This conceptual approach could be key to determining the Ð*_fA_* value; however, it is likely applicable only to chitosan samples that can exhibit complete solvation and ionization. This ideal scenario is unlikely for conventional chitosan samples, as ionization has been shown to be an asymptotic process [74]. Adjusting the ionic strength of the medium to achieve high ionization leads to aggregation or coiling of macromolecules, thereby altering charge density by ceasing to be linear. However, it would be possible to obtain a pseudo-Ð*_fA_*, since samples with a high molecular weight and degrees of ionization near 1 could not imply a significant change in the linear charge density, and macromolecules with close *f_A_* (not equal) could be separated.

By presenting this conceptual framework, we provide a potential roadmap for future investigations into Ð*_fA_*. Clarifying this parameter is essential to advancing in the characterization of these biopolymers.

## 6. Conclusions

“Chitosan” is a term that encompasses a broad spectrum of molecular configurations that can be identified through three characterization parameters: *f_A_*, *X*, and *P_A_*. However, these parameters are descriptive statistical values obtained from a set of molecular species that present a dispersity; conventional chitosan samples are always composed of macromolecules with different molecular configurations. The findings of the present study suggest that dispersity in the degree of polymerization could lead to a low reproducibility of bioactivity results on the germination of maize seeds, potentially explaining the ambiguous results in the literature referring to the application of conventional chitosan to this cereal, and generating that only partial consensus on the biological activities observed can be extrapolated. The findings of the present study highlight the need to strengthen efforts toward processes capable of obtaining chitosans with low dispersity. Achieving greater molecular homogeneity is expected to enable more reproducible bioactivity research results and support the future development of reliable chitosan-based bioproducts. According to the findings of this work, it is recommended to report at least the Ð*_X_* when evaluating the biological activity of conventional chitosan, to inform readers that the reproducibility of the results could vary.

## Figures and Tables

**Table 1:** Characterization parameters of the chitosan samples.

Code	Source	*f_A_* ^a,g^	*X_w_* ^b,h^	Ð*_X_* ^c,h^	Classification
419419	Industrial	0.30	11,589	2.53	HMW ^d^
448877	Industrial	0.35	8372	2.46	MMW ^e^
448869	Industrial	0.22	4518	2.47	LMW ^f^
Q16H	Laboratory	0.16	17,161	1.80	HMW ^d^
Q25H	Laboratory	0.25	15,563	1.63	
Q33H	Laboratory	0.33	13,334	1.60	
Q09M	Laboratory	0.09	10,231	2.25	MMW ^e^
Q24M	Laboratory	0.24	9834	2.16	
Q32M	Laboratory	0.32	9134	2.22	
Q07L	Laboratory	0.07	5703	1.87	LMW ^f^
Q23L	Laboratory	0.23	5558	1.81	
Q27L	Laboratory	0.27	5821	1.87	

^a^ Molar fraction of acetylation. ^b^ Weight-average degree of polymerization. ^c^ Dispersity of the degree of polymerization. ^d^ High molecular weight. ^e^ Medium molecular weight. ^f^ Low molecular weight. ^g^ Analytical precision for *f_A_* was ± 0.0035, as determined by duplicate ^1^H NMR analysis of sample 419419. ^h^ For *X*_w_ and Ð_X_, an instrumental error of 5% (relative standard deviation) was assumed, which is the standard acceptable margin for relative GPC/SEC characterization of polydisperse polymers [25,26].

**Table 2:** Partial consensus and particularities on the in vitro application of conventional chitosans in the germination of maize seeds.

Partial Consensus (General Trends)	Particularities
❖ Larger degrees of polymerization induce a more significant inhibition of germination. This inhibition is more pronounced as the concentration increases in the range of medium to high molecular weight.	❖ HMW and LMW subsets present a more significant inhibition at the middle values of *f_A_*, and the MMW subset at the lower value. Correlation with other parameters is necessary because the reported molar fraction of acetylation and degrees of polymerization values are averages.
❖ The application of chitosan delays the germination process in the range of low to high molecular weight. This delay increases with the chitosan concentration.	
❖ For all subsets (HMW, MMW, and LMW), the average root length decreases with the application of chitosan, and this trend is exacerbated as the degree of polymerization and concentration increase.	❖ The Q07L sample shows an almost null tendency to slow down the germination rate, a trend not observed in the same subset’s other samples (Q23L and Q27L). As this subset is the most homogeneous in its degree of polymerization, it allows us to presuppose that the molar fraction of acetylation effectively causes an effect on the germination rate. Discrepancies observed could be correlated with the intermolecular dispersity of the molar fraction of acetylation, a parameter that could not be determined for this study.
❖ For all subsets (HMW, MMW, and LMW), the average epicotyl length decreases with the application of chitosan, and this trend is exacerbated as the degree of polymerization and concentration increase.	

**Table 3:** Degrees of ionization estimated using the model proposed by Giraldo et al. (2021) [74].

**C_Cht_^a^ (% w)**		**LMW**			**MMW**			**HMW**		
		**Q07L**	**Q23L**	**Q27L**	**Q09M**	**Q24M**	**Q32M**	**Q16H**	**Q25H**	**Q33H**
	***f*_A_**	0.07	0.23	0.27	0.09	0.24	0.32	0.16	0.25	0.33
	***X***	5703	5558	5821	10,231	9834	9134	17,161	15,563	13,334
	**Average degree of ionization (β) ^b^**									
0.25		0.64	0.61	0.60	0.64	0.61	0.59	0.63	0.61	0.59
0.50		0.68	0.67	0.67	0.68	0.67	0.66	0.67	0.67	0.66
0.75		0.69	0.68	0.68	0.69	0.68	0.68	0.69	0.68	0.68
1.00		0.68	0.69	0.69	0.68	0.69	0.69	0.68	0.69	0.69

^a^ Chitosan concentration. ^b^ Degree of ionization at pH 5.

## Data Availability

The original contributions presented in this study are included in the article/Supplementary Materials. Further inquiries can be directed to the corresponding author.

## Supplementary Materials

The following supporting information can be downloaded at: https://www.mdpi.com/article/10.3390/polym18091032/s1, Figure S1: Effect on maize seeds biomass at day 8 following treatment with chitosan solutions of (A) high molecular weight (*X*: 15,353 ± 1922), (B) medium molecular weight (*X*: 9733 ± 556), and (C) low molecular weight (*X*: 5694 ± 131). Different letters indicate significant differences among concentrations according to Tukey’s test at *p* ≤ 0.05; Figure S2: Effect on maize seeds biomass following treatment with chitosan solutions of (A) high molecular weight (*X*: 15,353 ± 1922), (B) medium molecular weight (X: 9733 ± 556), and (C) low molecular weight (*X*: 5694 ± 131); Table S1: Research on the bioactivity of chitinous materials in the seed germination of several plant species; Table S2: Repeatability and reproducibility (R&R) analysis of variance (ANOVA) for sample 419419; Table S3: Repeatability and reproducibility (R&R) analysis of variance (ANOVA) for sample Q07L. References [4,5,6,7,8,10,12,63,64,65,66,79,80,81,82,83,84,85,86,87,88,89,90,91,92,93,94,95,96,97,98,99] are cited in Supplementary Materials.
