# Supplementary material for: Beyond Averages: Chitosan Dispersity Affects the Bioactivity Reproducibility on In Vitro Maize (var. Jubilee) Germination
Source: Polymers (Basel). 2026 Apr 24;18(9):1032. doi: 10.3390/polym18091032 (PMC13165036; doi:10.3390/polym18091032)
Supplement: Supplementary file 1 [file polymers-18-01032-s001.zip › polymers-4241202-supplementary.pdf]

Supporting Information

Beyond Averages: Chitosan Dispersity Affects the Bioactivity Reproducibility on *In Vitro* Maize (*var. Jubilee)* Germination

Juan D. Giraldo[a*], Ariana F. Peña[b], Claudia B. Briceño[c], Daniela Andrade-Acuña[c], Luis Aranibar[a], Karla A. Garrido-Miranda[d], Mauricio Schoebitz[b]

juan.giraldo@uach.cl

[a]*Laboratorio de Investigación en Biopolímeros (LIB), Escuela de Ingeniería Ambiental, Instituto de Acuicultura, Universidad Austral de Chile, Sede Puerto Montt, Balneario Pelluco, Los Pinos s/n, Chile. luis.aranibar@uach.cl*

[b]*Departamento de Suelos y Recursos Naturales, Facultad de Agronomía, Campus Concepción, Casilla 160-C, Universidad de Concepción, Chile. arpena2018@udec.cl (A.F.P.); mshoebitz@udec.cl (M.S.)*

[c]*Centro Superior de Ciencias Básicas, Universidad Austral de Chile, Sede Puerto Montt, Balneario Pelluco, Los Pinos s/n, Chile. claudia.briceno@uach.cl (C.B.B.); daniela.andrade@uach.cl (D.A.-A.)*

[d]*Center of Waste Management and Bioenergy, Scientific and Technological Bioresource Nucleus, BIOREN-UFRO, Universidad de la Frontera, Temuco, 4811230, Chile. karla.garrido@ufrontera.cl*

Supporting Information

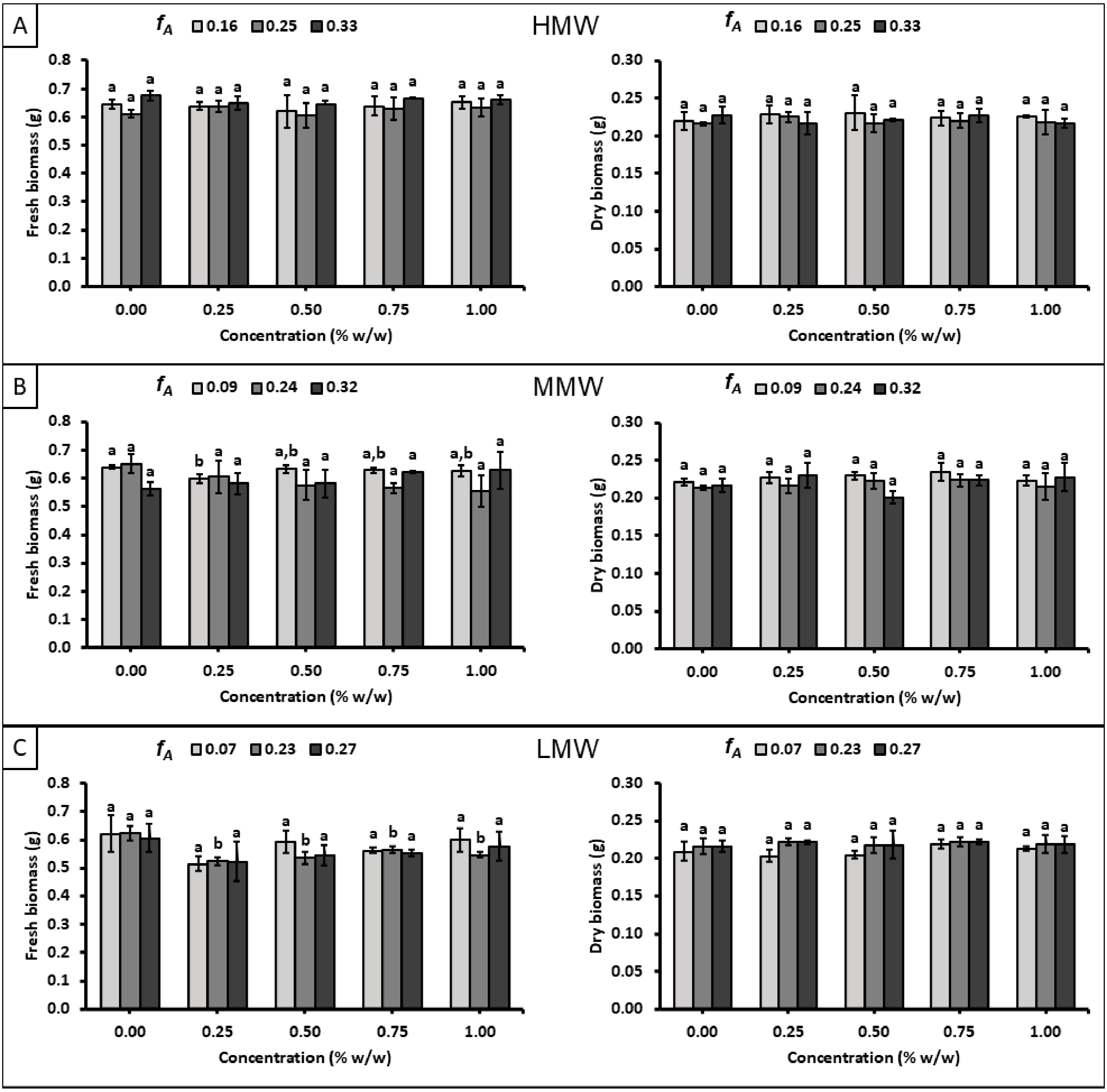

Figure S1. Effect on maize seeds biomass at day 8 following treatment with chitosan solutions of A) high molecular weight (X: 15353 ± 1922), B) medium molecular weight (X: 9733 ± 556), and C) low molecular weight (X: 5694 ± 131). Different letters indicate significant differences among concentrations according to Tukey's test at $p \leq 0.05$.

Supporting Information

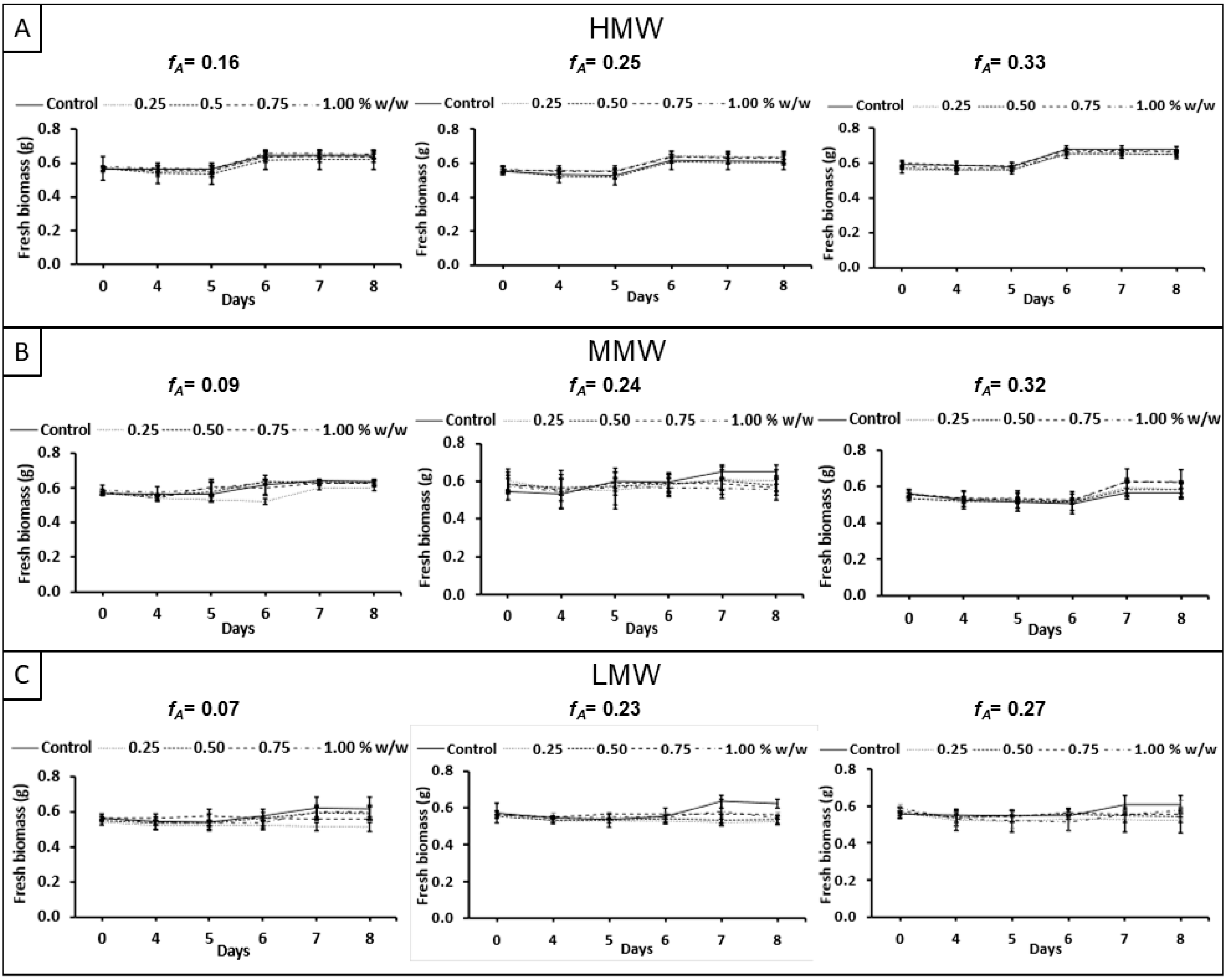

Figure S2. Effect on maize seeds biomass following treatment with chitosan solutions of A) high molecular weight (X: 15353 ± 1922), B) medium molecular weight (X: 9733 ± 556), and C) low molecular weight (X: 5694 ± 131).

Table S1. Research on the bioactivity of chitinous materials in the seed germination of several plant species.

N°	Title	M_w (g/mol)	X	f_A	Supplier	Results*	Seed
1	Application of enzymatic hydrolyzate of chitosan as a plant growth promoter (2023) [1]	1,000-30,000	-	0.05-0.25	Bioprogress LLC	P	Pea and Cucumber
2	The effects of chitosan and its acetylation degree on *in vitro* seed germination and organ development in *Ageratum houstonianum* Mill (2022) [2]	329-2,468<br>336-2,522	2-15	0.10<br>0.20	Institute of Plant Biology and Biotechnology, University of Münster	A and Nu	Blue billygoat weed
3	Chitosan coating on bean and maize seeds: release of agrochemical fungicide and post-storage condition (2021) [3]	-	-	-	Own-source**	Nu	Bean and Maize
4	Seed priming with copper-loaded chitosan nanoparticles promotes early growth and enzymatic antioxidant defense of maize (*Zea mays L.*) seedlings (2021) [4]	LMW***	-	0.25	Sigma-Aldrich	A	Maize
5	Chitosan-silicon nanofertilizer to enhance plant growth and yield in maize (*Zea mays L.*) (2021) [5]	50,000-190,000	74-1,130	0.20	Sigma-Aldrich	P	Maize
6	Decontamination of seeds destined for edible sprout production from *Listeria* by using chitosan coating with synergetic lysozyme-nisin mixture (2020) [6]	50,000-190,000<br>190,000-300,000<br>310,000-375,000	-	0.15-0.25	Sigma-Aldrich	Nu and A	Bean, Lentil and Wheat
7	Biogenic synthesis of iron oxide nanoparticles using *Moringa oleifera* and chitosan and its evaluation on corn germination (2020) [7]	-	-	-	-	P	Maize
8	Treatment of bell pepper (*Capsicum annuum L.*) seeds with chitosan increases chitinase and glucanase activities and enhances emergence in a standard cold test (2020) [8]	-	-	-	Sigma-Aldrich	P	Bell pepper

9	Chitosan versus plant growth regulators: a comparative analysis of their effects on *in vitro* development of *Serapias vomeracea* (Burm.f.) Briq. (2020) [9]	329-2,468<br>11,519	2-15<br>70	0.10	Institute of Plant Biology and Biotechnology, University of Münster	P	Orchid
10	Effect of chitosan polymer and inoculated with *B. japonicum* on soybean germination survival of seedling, nodulation and bacteria viability on seeds (2019) [10]	LMW***	-	0.20	Sigma-Aldrich	P	Soybean
11	Effects of chitosan nanoparticles on seed germination and seedling growth of wheat (*Triticum aestivum L.*) (2019) [11]	22,000	136	0.03	Aladdin	P	Wheat
12	Preparation of chitooligosaccharide by hydrogen peroxide degradation of chitosan and its effect on soybean seed germination (2019) [12]	4,500	27	0.07	Own-source**	P	Soybean
13	Optimization of chitosan nanoparticle synthesis and its potential application as germination elicitor of *Oryza sativa L*. (2019) [13]	-	-	-	-	P	Rice
14	Development of polyvinyl alcohol/chitosan hydrogel loaded with fertilizer compound: preparation, properties and effect on seed germination (2018) [14]	-	-	-	Merck	P	Okra
15	Effects of bare and chitosan-coated Fe_3O_4 magnetic nanoparticles on seed germination and seedling growth of *Capsicum annuum L*. (2018) [15]	20,000	122	0.08	Amicogen Inc.	P and A	Sweet pepper
16	Chitosan nanoparticles having higher degree of acetylation induce resistance against pearl millet downy mildew through nitric oxide generation (2018) [16]	20,000	106	0.70	-	P	Pearl millet

17	Phytotoxicity of chitosan and SiO_2 nanoparticles to seed germination of wheat (*Triticum aestivum L.*) and Barley (*Hordeum vulgare L.*) plants (2017) [17]	-	-	-	-	P, Nu, and A	Wheat and Barley
18	Preparation of Cu-chitosan nanoparticle and its effect on growth and enzyme activity during seed germination in maize (2017) [18]	-	-	-	Sigma-Aldrich	Nu	Maize
19	Cu-chitosan nanoparticle mediated sustainable approach to enhance seedling growth in maize by mobilizing reserved food (2016) [19]	LMW***	-	0.20	Sigma-Aldrich	A	Maize
20	Pepper (*Capsicum annuum*) seed germination and vigour following nanochitin, chitosan or hydropriming treatments (2016) [20]	-	-	-	NanoAgro Center	P	Bell pepper
21	Solid matrix priming with chitosan enhances seed germination and seedling invigoration in mung bean under salinity stress (2016) [21]	-	-	-	-	P	Bean
22	Silver-chitosan nanoparticles induced biochemical variations of chickpea (*Cicer arietinum L.*) (2016) [22]	-	-	-	-	P	Chickpea
23	Chitosan effects on phytopathogenic fungi and seed germination of *Jatropha curcas L.* (2015) [23]	LMW***	-	-	Sigma-Aldrich	Nu	Jathrofa curcas
24	Application of chitin hydrogels for seed germination, seedling growth of rapeseed (2014) [24]	50,0000	2,493	0.95	Zhejiang Golden-Shell Biochemical Co.	Nu	Rape
25	Effect of chitosan coating on seed germination and salt tolerance of lentil (*Lens culinaris L.*) (2013) [25]	-	-	-	-	P	Lentil

26	Maize seed coatings and seedling sprayings with chitosan and hydrogen peroxide: their influence on some phenological and biochemical behaviors (2013) [26]	125,000	763	0.08	Own-source**	Nu	Maize
27	Studies on appropriate chitosan type and optimum concentration on rice seed storability (2013) [27]	-	-	-	-	P	Rice
28	Chitosan application in maize (*Zea mays*) to counteract the effects of abiotic stress at seedling level (2011) [28]	1836	11	0.05	Own-source**	Nu	Maize
29	Application of bioactive coatings based on chitosan for artichoke seed protection (2010) [29]	149,000<br>400,000	-	-	Marinard Biotech	P	Artichoke
30	Seed priming with chitosan improves maize germination and seedling growth in relation to physiological changes under low temperature stress (2009) [30]	-	-	-	Yuhuan Chemicals	Nu	Maize
31	Chitosan treatment of wheat seeds induces resistance to *Fusarium graminearum* and improves seed quality (1999) [31]	-	-	-	Nova-Chem	P	Wheat
32	Chitosan benefits cultivation of vegetables (1998) [32]	-	-	0.3<br>0.2<br>0.1	Own-source**	Nu	Alfalfa, Wheat, Pea, Bean and Raddish

*Only refers to results on seed germination following treatment with a chitinous material: Positive (P), null (Nu), or adverse (A) effect relative to a control.
**Refers to samples obtained by the authors in their laboratories.
***Low molecular weight.
- Not reported.

Supporting Information

Table S2. Repeatability and reproducibility (R&R) analysis of variance (ANOVA) for sample 419419.

ANOVA				Alpha	0.05	
	SS	*df*	*MS*	*F*	*p-value*	*sig*
Part	171399.81	19	9021.0428	48.35872413	3.313E-12	
Operator	81.10724	1	81.10724	0.434788166	0.5175619	
Operator*Part	3544.3411	19	186.54427	1.997238083	0.017476	yes
Repeatability	7472.0894	80	93.401117			
Total	182497.35	119	1533.5912			
VARIATION						
	Var	*%*	*Std Dev*	*%*		
Tot Gage R&R	124.44883	7.8%	11.155664	27.9%		
- Repeatability	93.401117	5.8%	9.6644253	24.2%		
- Reproducibility	31.047717	1.9%	5.5720478	13.9%		
-- Operator	0	0.0%	0	0.0%		
-- Op*Part	31.047717	1.9%	5.5720478	13.9%		
Part-to-Part	1472.4164	92.2%	38.372079	96.0%		
Tot Variation	1596.8653	100.0%	39.960796	100.0%		
No. of categories	4					

Table S3. Repeatability and reproducibility (R&R) analysis of variance (ANOVA) for sample Q07L.

ANOVA				Alpha	0.05	
	SS	*df*	*MS*	*F*	*p-value*	*sig*
Part	191687.58	19	10088.82	414.3423949	6.201E-21	
Operator	249.30044	1	249.30044	10.23863479	0.0047164	
Operator*Part	462.63086	19	24.348993	2.673959653	0.0011942	yes
Repeatability	728.47749	80	9.1059686			
Total	193127.99	119	1622.9243			
VARIATION						
	Var	*%*	*Std Dev*	*%*		
Tot Gage R&R	17.936167	1.1%	4.2351113	10.3%		
- Repeatability	9.1059686	0.5%	3.0176098	7.3%		
- Reproducibility	8.8301989	0.5%	2.9715651	7.2%		
-- Operator	3.7491908	0.2%	1.9362827	4.7%		
-- Op*Part	5.081008	0.3%	2.2541091	5.5%		
Part-to-Part	1677.4118	98.9%	40.956218	99.5%		
Tot Variation	1695.348	100.0%	41.174604	100.0%		
No. of categories	13					

Supporting Information

REFERENCES

(1) Kotlyar, M.; Ibatullina, D.; Zinov'Eva, M.; Shnaider, K.; Zaripova, S. Application of Enzymatic Hydrolyzate of Chitosan as a Plant Growth Promoter. *E3S Web of Conferences* 2023, *443*, 3–8. https://doi.org/10.1051/e3sconf/202344301002.

(2) Gün Polat, E.; Acemi, A.; Özen, F. The Effects of Chitosan and Its Acetylation Degree on in Vitro Seed Germination and Organ Development in Ageratum Houstonianum Mill. *Plant Cell Tissue Organ Cult* 2022, *149* (3), 809–821. https://doi.org/10.1007/s11240-022-02305-5.

(3) Godínez-Garrido, N. A.; Ramírez-Pimentel, J. G.; Covarrubias-Prieto, J.; Cervantes-Ortiz, F.; Pérez-López, A.; Aguirre-Mancilla, C. L. Chitosan Coating on Bean and Maize Seeds: Release of Agrochemical Fungicide and Post-Storage Condition. *Journal of Seed Science* 2021, *43*. https://doi.org/10.1590/2317-1545v43254286.

(4) Gomes, D. G.; Pelegrino, M. T.; Ferreira, A. S.; Bazzo, J. H. B.; Zucareli, C.; Seabra, A. B.; Oliveira, H. C. Seed Priming with Copper-Loaded Chitosan Nanoparticles Promotes Early Growth and Enzymatic Antioxidant Defense of Maize (Zea Mays L.) Seedlings. *Journal of Chemical Technology and Biotechnology* 2021, *96* (8), 2176–2184. https://doi.org/10.1002/jctb.6738.

(5) Kumaraswamy, R. V.; Saharan, V.; Kumari, S.; Chandra Choudhary, R.; Pal, A.; Sharma, S. S.; Rakshit, S.; Raliya, R.; Biswas, P. Chitosan-Silicon Nanofertilizer to Enhance Plant Growth and Yield in Maize (Zea Mays L.). *Plant Physiology and Biochemistry* 2021, *159*, 53–66. https://doi.org/10.1016/j.plaphy.2020.11.054.

(6) Sozbilen, G. S.; Yemenicioğlu, A. Decontamination of Seeds Destined for Edible Sprout Production from Listeria by Using Chitosan Coating with Synergetic Lysozyme-Nisin Mixture. *Carbohydr Polym* 2020, *235* (September 2019). https://doi.org/10.1016/j.carbpol.2020.115968.

(7) Tovar, G. I.; Briceño, S.; Suarez, J.; Flores, S.; González, G. Biogenic Synthesis of Iron Oxide Nanoparticles Using Moringa Oleifera and Chitosan and Its Evaluation on Corn Germination. *Environ Nanotechnol Monit Manag* 2020, 100350. https://doi.org/10.1016/j.enmm.2020.100350.

(8) Samarah, N. H.; AL-Quraan, N. A.; Massad, R. S.; Welbaum, G. E. Treatment of Bell Pepper (Capsicum Annuum L.) Seeds with Chitosan Increases Chitinase and Glucanase Activities and Enhances Emergence in a Standard Cold Test. *Sci Hortic* 2020, *269* (March), 109393. https://doi.org/10.1016/j.scienta.2020.109393.

(9) Acemi, A. Chitosan versus Plant Growth Regulators: A Comparative Analysis of Their Effects on in Vitro Development of Serapias Vomeracea (Burm.f.) Briq. *Plant Cell Tissue Organ Cult* 2020, *141* (2), 327–338. https://doi.org/10.1007/s11240-020-01789-3.

(10) Costales, D.; Nápoles, M. C.; Alejandro, F. R.; Gustavo, G. A.; Cecilia, P.; Susana, S.; Perrig; Diego. Effect of Chitosan Polymer and Inoculated with b. Japonicum on Soybean Germination Survival of Seedling, Nodulation and Bacteria Viability on Seeds. *Legume Research* 2019, *42* (2), 265–269. https://doi.org/10.18805/LR-410.

(11) Li, R.; He, J.; Xie, H.; Wang, W.; Bose, S. K.; Sun, Y.; Hu, J.; Yin, H. Effects of Chitosan Nanoparticles on Seed Germination and Seedling Growth of Wheat (Triticum Aestivum L.). *Int J Biol Macromol* 2019, *126*, 91–100. https://doi.org/10.1016/j.ijbiomac.2018.12.118.

(12) Hai, N. T. T.; Thu, L. H.; Nga, N. T. T.; Hoa, T. T.; Tuan, L. N. A.; Van Phu, D.; Hien, N. Q. Preparation of Chitooligosaccharide by Hydrogen Peroxide Degradation of Chitosan and Its Effect on Soybean Seed Germination. *J Polym Environ* 2019, *27* (9), 2098–2104. https://doi.org/10.1007/s10924-019-01479-y.

(13) Divya, K.; Vijayan, S.; Nair, S. J.; Jisha, M. S. Optimization of Chitosan Nanoparticle Synthesis and Its Potential Application as Germination Elicitor of Oryza Sativa L. *Int J Biol Macromol* 2019, *124*, 1053–1059. https://doi.org/10.1016/j.ijbiomac.2018.11.185.

(14) Hasraf, N.; Nayan, M.; Syahir, M.; Hamzah, A.; Mohd, A. A.; Anis, A.; Rajali, A.; Muslih, E. F.; Mazlan, R. Development of Polyvinyl Alcohol/Chitosan Hydrogel Loaded with Fertilizer Compound: Preparation, Properties and Effect on Seed Germination. *Journal of Science and Technology* 2018, *10* (4), 21–27.

(15) Bahrami, M.; Movafeghi, A.; Mahdavinia, G.; Hassanpouraghdam, M.; Gohari, G. Effects of Bare and Chitosan-Coated Fe3O4 Magnetic Nanoparticles on Seed Germination and Seedling Growth of Capsicum Annuum L. *Biointerface Res Appl Chem* 2018, *8* (5), 3552–3559.

(16) Siddaiah, C. N.; Prasanth, K. V. H.; Satyanarayana, N. R.; Mudili, V.; Gupta, V. K.; Kalagatur, N. K.; Satyavati, T.; Dai, X. F.; Chen, J. Y.; Mocan, A.; Singh, B. P.; Srivastava, R. K. Chitosan Nanoparticles Having Higher Degree of Acetylation Induce Resistance against Pearl Millet Downy Mildew through Nitric Oxide Generation. *Sci Rep* 2018, *8* (1), 1–14. https://doi.org/10.1038/s41598-017-19016-z.

(17) Behboudi, F.; Tahmasebi Sarvestani, Z.; Kassaee, M. Z.; Modares Sanavi, S. A. M.; Sorooshzadeh, A. Phytotoxicity of Chitosan and SiO2 Nanoparticles

to Seed Germination of Wheat (Triticum Aestivum L.) and Barley (Hordeum Vulgare L.) Plants. *Not Sci Biol* 2017, *9* (2), 242–249. https://doi.org/10.15835/nsb9210075.

(18) Choudhary, R. C.; Joshi, A.; Kumari, S.; Rv, K.; Saharan, V. Preparation of Cu-Chitosan Nanoparticle and Its Effect on Growth and Enzyme Activity during Seed Germination in Maize. *J Pharmacogn Phytochem* 2017, *6* (4), 669–673.

(19) Saharan, V.; Kumaraswamy, R. V.; Choudhary, R. C.; Kumari, S.; Pal, A.; Raliya, R.; Biswas, P. Cu-Chitosan Nanoparticle Mediated Sustainable Approach To Enhance Seedling Growth in Maize by Mobilizing Reserved Food. *J Agric Food Chem* 2016, *64* (31), 6148–6155. https://doi.org/10.1021/acs.jafc.6b02239.

(20) Samarah, N. H.; Wang, H.; Welbaum, G. E. Pepper (Capsicum Annuum) Seed Germination and Vigour Following Nanochitin, Chitosan or Hydropriming Treatments. *Seed Science and Technology* 2016, *44* (3), 609–623. https://doi.org/10.15258/sst.2016.44.3.18.

(21) Sen, S. K.; Mandal, P. Solid Matrix Priming with Chitosan Enhances Seed Germination and Seedling Invigoration in Mung Bean under Salinity Stress. *Journal of Central European Agriculture* 2016, *17* (3), 749–762. https://doi.org/10.5513/JCEA01/17.3.1773.

(22) Anusuya, S.; Banu, K. N. Silver-Chitosan Nanoparticles Induced Biochemical Variations of Chickpea (Cicer Arietinum L.). *Biocatal Agric Biotechnol* 2016, *8*, 39–44. https://doi.org/10.1016/j.bcab.2016.08.005.

(23) Pabón-Baquero, D.; Velázquez-Del Valle, M. G.; Evangelista-Lozano, S.; León-Rodriguez, R.; Hernández-Lauzardo, A. N. Chitosan Effects on Phytopathogenic Fungi and Seed Germination of Jatropha Curcas L. *Revista Chapingo, Serie Ciencias Forestales y del Ambiente* 2015, *21* (3), 241–253. https://doi.org/10.5154/r.rchscfa.2014.10.051.

(24) Tang, H.; Zhang, L.; Hu, L.; Zhang, L. Application of Chitin Hydrogels for Seed Germination, Seedling Growth of Rapeseed. *J Plant Growth Regul* 2014, *33* (2), 195–201. https://doi.org/10.1007/s00344-013-9361-5.

(25) Al-Tawaha, A. R. M.; Al-Ghzawi, A. L. A. Effect of Chitosan Coating on Seed Germination and Salt Tolerance of Lentil (Lens Culinaris L.). *Research on Crops* 2013, *14* (2), 489–491.

(26) Lizárraga-Paulín, E.-G.; Miranda-Castro, S.-P.; Moreno-Martínez, E.; Lara-Sagahón, A.-V.; Torres-Pacheco, I. Maize Seed Coatings and Seedling Sprayings with Chitosan and Hydrogen Peroxide: Their Influence on Some

Phenological and Biochemical Behaviors. *J Zhejiang Univ Sci B* 2013, *14* (2), 87–96. https://doi.org/10.1631/jzus.B1200270.

(27) Suvannasara, R.; Boonlertnirun, S. Studies on Appropriate Chitosan Type and Optium Concentration on Rice Seed Storability. *J Agric Biol Sci* 2013, *8* (3), 196–200.

(28) Lizárraga-Paulí, E. G.; Torres-Pacheco, I.; Moreno-Martínez, E.; Miranda-Castro, S. P. Chitosan Application in Maize (Zea Mays) to Counteract the Effects of Abiotic Stress at Seedling Level. *Afr J Biotechnol* 2011, *10* (34), 6439–6446. https://doi.org/10.5897/AJB10.1448.

(29) Ziani, K.; Ursúa, B.; Maté, J. I. Application of Bioactive Coatings Based on Chitosan for Artichoke Seed Protection. *Crop Protection* 2010, *29* (8), 853–859. https://doi.org/10.1016/j.cropro.2010.03.002.

(30) Guan, Y.; Hu, J.; Wang, X.; Shao, C. Seed Priming with Chitosan Improves Maize Germination and Seedling Growth in Relation to Physiological Changes under Low Temperature Stress. *J Zhejiang Univ Sci B* 2009, *10* (6), 427–433. https://doi.org/10.1631/jzus.B0820373.

(31) Reddy, M. V. B.; Arul, J.; Angers, P.; Couture, L. Chitosan Treatment of Wheat Seeds Induces Resistance to Fusarium Graminearum and Improves Seed Quality. *J Agric Food Chem* 1999, *47* (3), 1208–1216. https://doi.org/10.1021/jf981225k.

(32) Li, C. F.; Wu, J. C. Chitosan Benefits Cultivation of Vegetables. In *Advances in Chitin Science, Vol. 3, Proceedings of the 3rd Asia-Pacific Chitin and Chitosan Symposium*; Chen, R. H., Chen, H. C., Eds.; European Chitin Society: Keelung, 1998; pp 448–452.
